# Supplementary material for: Probing 10 μK stability and residual drifts in the cross-polarized dual-mode stabilization of single-crystal ultrahigh-Q optical resonators
Source: Light Sci Appl. 2019 Jan 3;8:1. doi: 10.1038/s41377-018-0109-7 (PMC6318213; doi:10.1038/s41377-018-0109-7)
Supplement: Supplementary file 1 — Supplemental Material [file 41377_2018_109_MOESM1_ESM.docx]

Supplementary Information

**Probing 10 μK stability and residual drifts in the cross-polarized dual-mode stabilization of single-crystal ultrahigh-*Q* optical resonators**

Jinkang Lim^1,*^, Wei Liang^2^, Anatoliy A. Savchenkov^2^, Andrey B. Matsko^2,*^, Lute Maleki^2^, and Chee Wei Wong^1,*^

* Corresponding authors: jklim001@ucla.edu; andrey.matsko@oewaves.com; cheewei.wong@ucla.edu

^1^ Fang Lu Mesoscopic Optics and Quantum Electronics Laboratory, University of California, Los Angeles, CA 90095 USA

^2^ OEwaves Inc., 465 North Halstead Street, Suite 140, Pasadena, CA 91107 USA

**S1. Mode extinction at each polarization mode**

There are no pure TM and TE modes but there exist TM-like and TE-like modes. In order to check the purities of the modes, we simulated the both TM and TE modes at each eigenfrequency of the whispering-gallery resonator and the power extinction ratios between TM and TE modes are $\approx$35 dB, which is pure enough, so that we ignore modal interaction contributions from the other residual mode at each eigenfrequency.


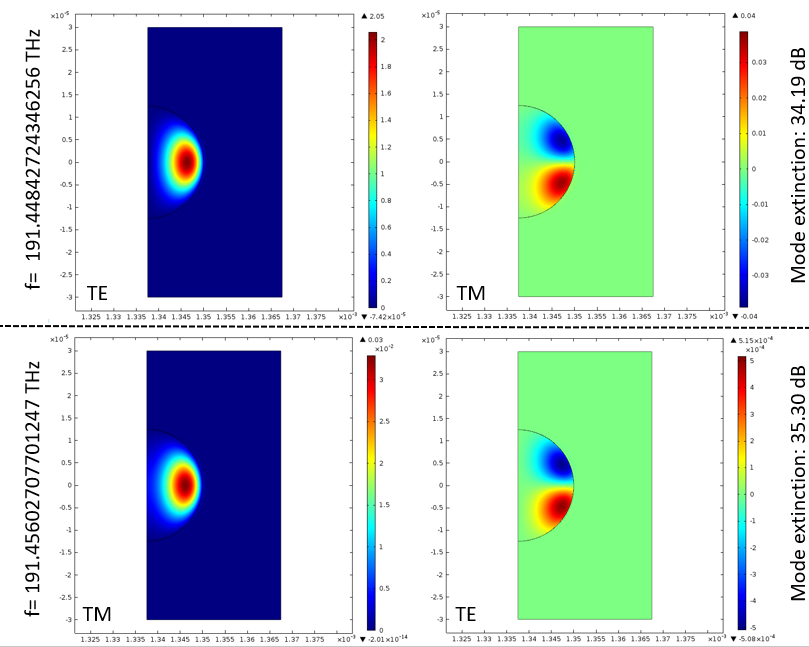


**Figure S1.** **TM and TE modes extinctions.** The mode extinction for both eigenfrequencies are $\approx$35 dB.

**S2. Impact of the modal area change due to the thermal expansion**

The change in the waveguide dimension due to the thermal expansion could introduce some changes in the size of modal areas (*A*_m_) and mode volumes (*V*_m_). To estimate this effect, we performed the FEM simulation and derived the change in *A*_m._ The different thermal expansion coefficients can change the waveguide shape asymmetrically (i.e. elliptic). The 1 K temperature variation changes the length perpendicular to the optical axis by 222.5 pm and the length parallel to the optical axis by 342.5 pm in the modal area. The deformation is small enough if we consider that our stabilized resonator mode volume temperature is controlled at 10 μK and thus it will not make meaningful impact on the size of the modal area and therefore on the power spectral density of the thermorefractive frequency noise.


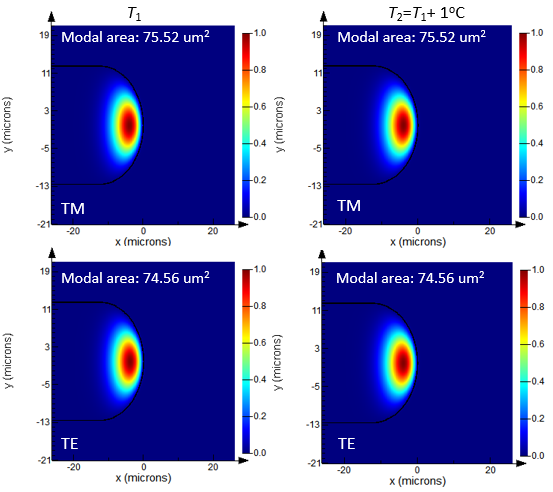


**Figure S2. FEM simulation of modal area variation due to the thermal expansion.** The different thermal expansion coefficient can change the waveguide shape asymmetrically (ellipse). The 1 K temperature variation changes the resonator length perpendicular to the optical axis by 222.5 pm and the resonator length parallel to the optical axis by 342.5 pm in the modal area. The deformation is small enough and does not make meaningful impact on the size of the modal area in numerical simulations.

**S3. Impact of ambient pressure**

The microresonator is placed in a compact vacuum chamber and evacuated to eliminate convective heat transfer and ambient perturbations such as pressure and humidity. The strong acoustic noise peaks above 1-kHz offset frequency are attenuated or eliminated, which is not compensated by the active dual-mode temperature stabilization feedback loop.


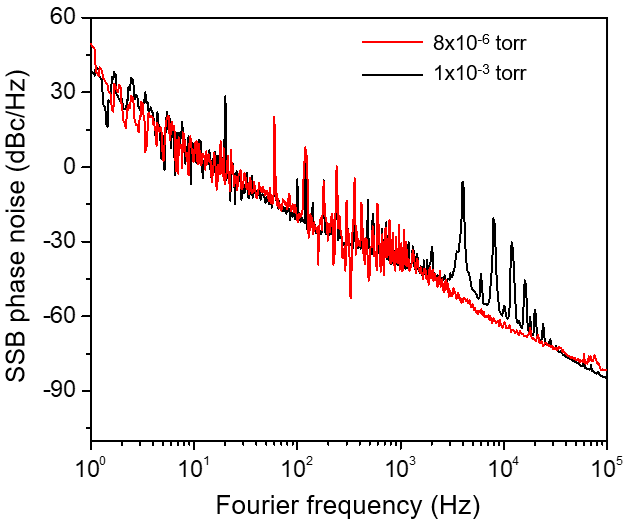


**Figure S3. SSB phase noise of the dual-mode beat frequency in different pressure levels.** Red line at 8×10^-6^ torr and black line at 1×10^-3^ torr. Acoustic noise is further suppressed at the lower pressure level.

**S4.** **Heat diffusion in the resonator and the resonator volume temperature distribution**

The absorbed light by the resonator can change not only local mode volume temperature but also change the resonator volume temperature via heat diffusion. We simulate the heat diffusion in the resonator caused by a laser heat source with the time-dependent heat diffusion analysis tool in the COMSOL Multiphysics, in which we exclude the contribution of independently feedback-controlled TEC. The elliptic-shaped heat source is centered at the TE mode peak with $\boldsymbol{\approx}$10 μm^2^ because most laser energy is concentrated near the mode peak as illustrated in Figure S4a and the 2D axis-symmetry geometry are implemented for simulating the cylindrical whispering-gallery resonator structure. We use parameters that use in the experiment for this simulation and they are shown in a table. The thermal conductivity of 21 W•m^-1^K^-1^, the density of 3180 kg• m^-3^ and heat capacity at constant pressure of 920 J•kg^-1^K^-1^ are used for the MgF_2_ resonator respectively. We calculate a characteristic diffusion time ($\boldsymbol{\approx}$30 sec) that provides the difference of the mode volume temperature (*T*_m_) and the averaged resonator volume temperature (*T*_c_) becomes a constant illustrated in Figure S4b and the result is also shown in Figure 3e. In the simulation, *T*_m_ is measured around the optical mode volume (circled area in Figure S4a) and *T*_c_ is measured for the entire resonator.


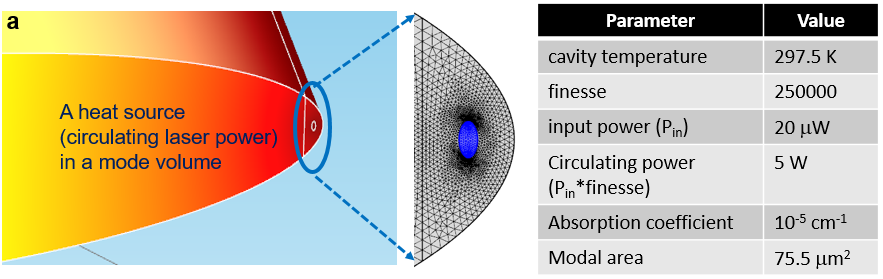


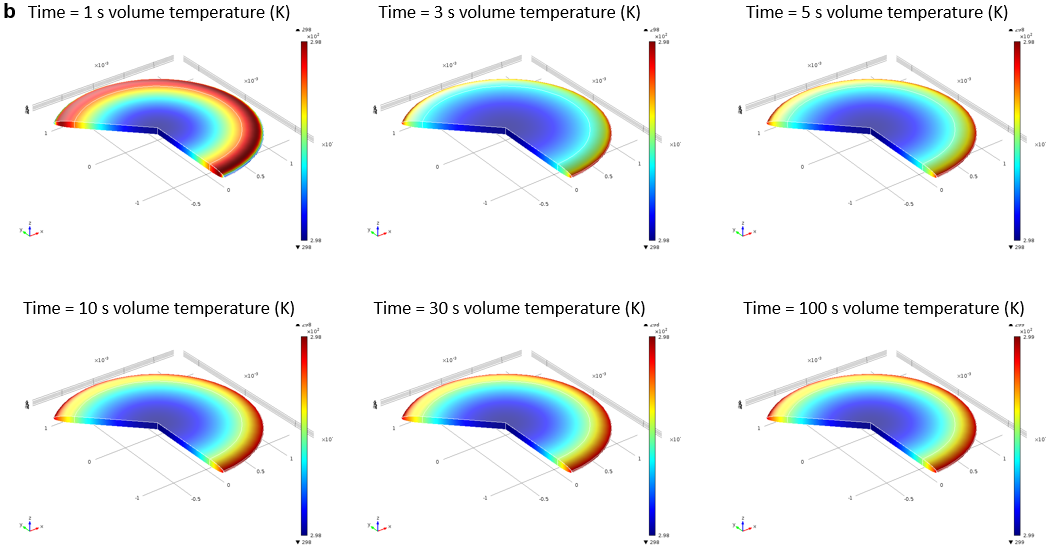


**Figure S4.** **FEM simulation of the heat diffusion in the resonator.** FEM simulation of the heat diffusion in the resonator and the resonator volume temperature distribution in time when a laser heat source is located at the center of the TE mode. The temperature distribution shows negligible changes after 30 s.

**S5. Impact of the relative intensity noise (RIN) on the resonator temperature stability**

To quantify the laser RIN contribution to the resonator temperature stability. We investigate a transfer function showing the resonance frequency shift induced by the laser intensity modulation. The coupled laser power is modulated by an acousto-optic modulator with a 1 Hz top-hat function with the modulated laser power (0.75 %) into the resonator and the corresponding TM laser frequency is measured. TM laser frequency shift is measured. We determine resonance frequency shift on the coupling power modulation – 2.66 kHz for 1 % coupled power change corresponding to 200 nW.


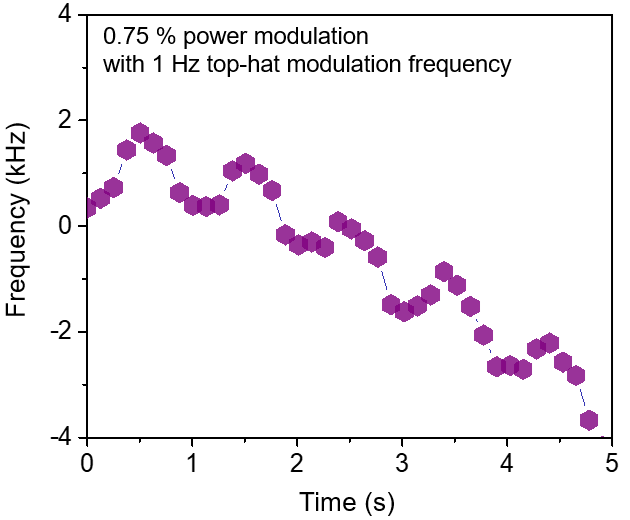


**Figure S5. The TM laser frequency shift due to the coupled laser intensity modulation.** The TM laser frequency response to the input power modulation. The TE laser is modulated with a 1 Hz top-hat function and the TM laser frequency response is measured to be 2 kHz for 0.75 % coupled power change.
